# Development of Cellulose Microfibers from Mixed Solutions of PAN-Cellulose in N-Methylmorpholine-N-Oxide

**DOI:** 10.3390/polym16131869

**Published:** 2024-06-30

**Authors:** Igor Makarov, Markel Vinogradov, Yaroslav Golubev, Ekaterina Palchikova, Yuriy Kulanchikov, Timofey Grishin

**Affiliations:** 1 A.V. Topchiev Institute of Petrochemical Synthesis, Russian Academy of Sciences, 29 Leninsky Prospect, 119991 Moscow, Russia; m.i.vinogradov1989@yandex.ru (M.V.); ya_golubev@ips.ac.ru (Y.G.); shatokhina@ips.ac.ru (E.P.); 2 Institute of Microelectronics Technology RAS, 142432 Chernogolovka, Russia; kulanchikov@iptm.ru; 3 Institute of Nanotechnology of Microelectronics of the Russian Academy of Sciences (INME RAS), Leninskiy Prospect, 32A, 119334 Moscow, Russia; grishin.t@outlook.com

**Keywords:** cellulose, PAN, N-methylmorpholine-N-oxide, fiber spinning, microfibers, mechanical properties

## Abstract

Mixed solutions of PAN with cellulose in N-methylmorpholine-N-oxide (NMMO) were prepared. Systems with a fraction of a dispersed phase of a cellulose solution in NMMO up to 40% are characterized by the formation of fibrillar morphology. The fibrils created as the mixed solution is forced through the capillary take on a more regular order as the cellulose content in the system drops. The systems’ morphology is considered to range from a heterogeneous two-phase solution to regular fibrils. The generated morphology, in which the cellulose fibrils are encircled by the PAN, can be fixed by spinning fibers. Cellulose fibrils have a diameter of no more than a few microns. The length of the fibrils is limited by the size of the fiber being formed. The process of selectively removing PAN was used to isolate the cellulose microfibrils. Several techniques were used to evaluate the mechanical properties of isolated cellulose microfibers. Atomic force microscopy allowed for the evaluation of the fiber stiffness and the creation of topographic maps of the fibers. Cellulose microfibers have a higher Young’s modulus (more than 30 GPa) than cellulose fibers formed in a comparable method, which affects the mechanical properties of composite fibers.

## 1. Introduction

With its distinct structure and characteristics, cellulose is one of the most abundant biopolymers on Earth [1]. It is produced by bacteria and certain animals, and it is an essential component of plant cell walls [2,3,4]. In addition to wood, crops including flax, hemp, miscanthus, nettle, etc. have proliferated as cellulose sources in recent years [5,6,7]. The availability of raw resources, together with the high cellulose and low lignin content of annual and herbaceous plants, justifies interest in them. Thus, flax and hemp have cellulose levels of 75 and 79%, respectively [8]. Wood generally contains less than 50% cellulose and up to 40% lignin [9].

The length of cellulose fibers varies depending on their source. For wood fibers, it is 1–2 mm; for cotton, the length reaches 6 cm, whereas the longest fibers are in bast crops, which can reach 1 m [10]. Unfortunately, the diameter of natural fibers varies greatly along the fiber. For man-made fibers, observed diameters have a smaller deviation from the average value. Long bast fibers, unlike wood fibers, require non-traditional cooking methods [11]. As a result, wood cellulose is still the most often used raw material for producing dissolving pulp [12].

Without concentrating on methods of pulping, which, by the way, make a significant contribution to the properties of the resulting polymer [13,14,15], we note that the use of cellulose extends to almost all areas of human activity [16,17].

Structural features of cellulose, namely the system of hydrogen bonds [18], high crystallinity [19], the form factor of supramolecular formations, etc., provide a range of unique properties. These include high thermal and chemical resistance, sorption, biodegradability, the presence of functional groups that make it possible to obtain cellulose derivatives, and mechanical properties, namely high strength. The maximum possible values of the elastic modulus of cellulose were first determined to be 90–120 GPa [20]. Further development of theoretical calculations showed that cellulose crystallites can have a strength of 25 GPa and an elastic modulus of 250 GPa [21]. Unfortunately, in real conditions, such values are not achieved, and, with good orientation, an elastic modulus can reach up to 70 GPa [22]. This significant difference in the elastic modulus from the theoretical limiting values is explained by the presence of crystalline and amorphous regions in the microfibrils. For natural fibers, the formation of defects during plant growth is usually observed along their axis, which is reflected in their mechanical characteristics, which vary over a wide range [10,23,24]. For man-made cellulose fibers, a more uniform structure is formed compared to natural fibers, which significantly reduces the spread of mechanical characteristics, but it is not possible to achieve a more ordered packing of macromolecules [4]. In the process of dissolution and regeneration of cellulose, macromolecules form a new system of hydrogen bonds and form microfibrils and microfibrillar aggregates with highly ordered and disordered regions [25]. As a result, the strength and elastic modulus values for regenerated fibers are lower compared to natural fibers, and the relative elongation, on the contrary, increases [26,27].

It is known that potential solvents for cellulose include solvents with high donor properties [28]. The works [29,30,31,32,33] studied the dissolution of cellulose in LiOH/urea/water, DMA/LiCl, sodium hydroxide, aqueous solutions of zinc chloride, and ionic liquids. The listed direct cellulose solvents have not found industrial application for several reasons, in particular due to the high cost and difficulty of regeneration.

Today, cellulose fibers are produced in commercial amounts only by viscose and NMMO processes [34,35,36]. The viscose process occupies a dominant position in the production of cellulose fibers; however, it is accompanied by the release of enormous amounts of hazardous compounds into the atmosphere. An alternative to the viscose method has been developed, the NMMO-process, which uses N-methylmorpholine-N-oxide (NMMO) as a cellulose solvent [37]. Traditional and solid-phase NMMO-processes can be distinguished based on the hydration form of NMMO [38,39]. The traditional process employs a 50% aqueous solution of NMMO from which water is gradually removed to the monohydrate form (13.3% water, T_m_ = 76 °C), which is a solvent for cellulose. The solid-phase process produces highly concentrated dopes in short periods of time by using a solvent containing less than 13% water.

The presence in the NMMO molecule of a semipolar N→O bond with two lone electron pairs on the oxygen atom ensures good solubility not only of cellulose but also of some synthetic polymers, including polyacrylonitrile (PAN) copolymers [40]. To obtain mixed solutions, it is proposed that a powder mixture of cellulose with PAN in NMMO be subjected to preliminary solid-phase activation and then melted (dissolved). The most suitable range of water content in the solvent is 8–13.3%. As the water content decreases, the melting point of NMMO/water systems and cellulose solubility increase.

The use of a meltable solvent determines the method of fiber spinning (the dry-jet wet method). The advantage of this spinning method is the possibility of drawing (orienting cellulose macromolecules) the resulting fiber into the air gap between the die and the precipitation bath. As a result, regenerated cellulose fibers have better strength characteristics and lower relative elongation compared to fibers obtained using the viscose process [41].

It was previously shown that the formation of mixed solutions based on cellulose and PAN in NNMO leads to the formation of fibrillar morphology [42]. The observed fibrils are formed due to the interaction between the molecules of the dispersed medium and cellulose, which acts as a dispersed phase. Using the method of selective dissolution of PAN, it was shown that microfibrils are formed by cellulose. Compared to nano- or microcrystalline cellulose, the diameter of which can reach 20 nm and 50 μm and the length of 100 nm and 1000 μm, respectively [43,44], cellulose microfibrils have an “unlimited” length. That is, cellulose can be considered a high-form filler.

There are numerous papers [45,46,47] in which it is shown that cellulose (its particles) can be used to increase the strength and rigidity of polymer composites. The mechanical properties of such composites will depend on the distribution of a cellulose additive in a polymer matrix and the geometry of the particles. With a uniform distribution of nano- and microcrystalline cellulose particles in a volume of a synthetic polymer, one can expect an increase in the strength and elastic modulus of composites based on them. The absence of agglomerates in the composite material prevents stress concentration in the polymer matrix, having a positive effect on the mechanical behavior of the composite.

When the length of cellulose fillers surpasses that of nano- and microcrystalline cellulose, the problem of homogeneous dispersion in a polymer matrix is not trivial. It was shown in [48] that an increase in longitudinal dimensions of the filler compared to “short” particles allows for greater rigidity of the composite. As the filler fraction increases, the difference between the observed values for systems based on “short” and “extended” fillers increases. Changing the shape of filler particles from short particles (l/d ≥ 1) to microfibrils of infinite length (with proper orientation) reduces the volume fraction of the filler while maintaining the same stiffness of the composite.

Based on the research reviewed above, we can conclude that the NMMO-process is the only true alternative to the viscose process, allowing us to produce mixed solutions and composite fibers from them. The cellulose phase’s shape and percentage will determine the mechanical properties of composite fibers, which can vary widely. The most interesting morphology is fibrillar, in which long microfibrils are formed by the elongated cellulose phase. This brings up the following scientific questions:

- How does deformation affect the fibrillar morphology of mixed solutions;
- Is it possible to obtain continuous cellulose microfibers using the dry-jet wet spinning method;
- What are the mechanical properties of cellulose fibrils separated from the matrix polymer;
- What role does the cellulose fibrillar phase play in composite fibers?

In the present study, therefore, composite fibers based on PAN and cellulose were formed from solutions in NMMO. The cellulose phase was separated from the PAN matrix using selective dissolving. Consequently, we discovered for the first time that cellulose may form continuous fibrils, which can be continuously produced and further extracted using aprotic PAN solvents like DMSO. The mechanical characteristics of the resulting microfibers were investigated. We hypothesized that the cellulose phase during the deformation of the mixed solution in NMMO forms fibrils that reinforce the PAN matrix, and their strength characteristics can exceed the values obtained for PAN fibers.

## 2. Materials and Methods

Mixed solutions based on PAN and cellulose in N-methylmorpholine-N-oxide (NMMO) with a total polymer content in the system of 18% were prepared using powdered cellulose (Baikal Pulp and Paper Mill, Baykalsk, Russia) (DP = 600, moisture content ~ 8%, mass content in the dry residue of α-cellulose ~ 92%, particle size ≤ 250 μm) and PAN ternary copolymer, the following composition: 93.9% acrylonitrile, 5.8% methyl acrylate, 0.3% methyl sulfonic acid ester, Mw = 85,000 g/mol, with an average particle size of 50 µm (Good Fellow, Huntingdon, UK).

A direct solvent of PAN and cellulose NMMO with a melting point of 100–120 °C (water content < 10%) was supplied by Demochem (Shanghai, China). Propyl gallate (0.5%) (Sigma-Aldrich, St. Louis, MI, USA) was introduced into the system to inhibit the processes of thermal-oxidative destruction of cellulose.

The solid-phase activation of ternary systems was used to obtain mixed solutions and composite fibers [49,50]. Cellulose and PAN in NMMO were activated under uniform compression and shear deformation conditions.

To obtain fluid solutions, the activated solid-phase pre-solutions were passed through the barrel of a HAAKE Minilab II twin-screw laboratory mixer (ThermoFisher Scientific, Dreieich, Germany) set to 120 °C and 100 rpm screw rotation.

The morphology of solutions and fibers was studied using polarization microscopy (Boetius microscope, VEB Kombinat Nadema, Germany and a Biomed 6PO microscope (Biomed Co., Moscow, Russia) equipped with a ToupTek E3ISPM5000 camera (ToupTek Photonics Co., Hangzhou, China)).

The composite fibers were formed using a dry-jet wet method on a Rheoscope 1000 viscometer (CEAST, Torino, Italy) with a receiving device at 120 °C. The mixed solution obtained on the twin-screw laboratory mixer was placed in the viscometer chamber and thermostated. Following that, the spinning solutions (viscosity slightly less than 10^3^ Pa*s at a shear rate of 0.01 s^−1^) were passed through a capillary with dimensions of 0.5 mm (diameter) and 5 mm (length) (an aspect ratio (l/d) of 10) at a temperature of 120 °C. The formed hot extrudate, after passing through a 15 cm air gap, fell into a precipitation water bath (T = 20 ± 2 °C). The resulting wet fibers were collected on a receiving shaft. After being removed from the shaft, the fibers were also washed in water and dried at room temperature.

To extract cellulose microfibers, the selective solvent PAN DMSO (Khimmed, Russia) was used. The composite fibers were immersed in a DMSO bath and left for 24 h at room temperature. Following this, a fresh solvent was used. The procedure was repeated at least three times.

The mechanical properties of the fibers were determined using an Instron 1122 tensile testing machine (Instron, Norwood, MA, USA) at a tensile speed of 10 mm/min with a base of 10 mm. Tests were carried out by GOST 10213.4–2002 [51]. For each sample, the number of tests was at least 20.

The morphology of the fibers’ surface and transverse cleavages was studied by low-voltage scanning electron microscopy (SEM) on an FEI Scios microscope (Hillsboro, OR, USA) with an accelerating voltage of less than 1 kV in the secondary electron mode.

The surface morphology of the fibers and the value of their Young’s modulus were studied using a Bruker Dimension Icon atomic force microscope (Bruker, Billerica, MA, USA). The sample was prepared as follows: using tweezers, a bundle of microfibers was extracted; with a second tweezer, the bundle was pulled out and separated until a few fibers remained between the two separated bundles. Next, the isolated microfibers were placed on a glass slide and dried to equilibrium moisture content. The scanning area was selected using an optical navigation microscope—scanning was carried out in places with the smallest observed diameter.

The AFM study of the mechanical properties of the fibers was carried out using the surface mechanical properties mapping mode PeakForce-QNM of Dimension Icon AFM. PeakForce-QNM is a sub-resonance tapping AFM mode in which an AFM probe oscillates quasi-harmonically up and down near the sample surface, tapping it in the lower part of its trajectory. Force curves (tip-sample interaction force versus displacement) are obtained in each cycle of these oscillations. Thus, an array of force data is captured. Using a probe with known stiffness, one can calculate tip-surface interaction force and obtain Young’s modulus values from force curves. According to [52], PeakForce-QNM can provide quantitative results provided that the Young’s modulus of the sample is in the range of 700 kPa–70 GPa, the probe is properly calibrated, and the DMT model (Derjagin, Muller, Toropov) [53] is applicable.

To obtain quantitative results utilizing the DMT model, one should know the deflection sensitivity of the cantilever, its spring constant, and its tip radius. There are two approaches to performing calibration: absolute and relative. Calibration according to the absolute method consists of measuring the deflection sensitivity on a hard sample, measuring the spring constant using thermal tuning, and estimating the tip radius using a blind test procedure [54]. To calibrate according to the relative method, one should use a sample with a known Young’s modulus as an etalon.

We used a silicon probe with a rectangular RTESPA-525 cantilever (Bruker, Billerica, MA, USA) with a nominal stiffness k = 200 N/m, a nominal resonant frequency of 525 kHz, and a nominal tip radius of 8 nm. To calculate Young’s modulus, the DMT model was used, since the radius of the probe rounding (~8 nm) exceeded the deformation depth (2–3 nm). For quantitative measurements, a relative calibration method was utilized with a fused quartz sample (E = 73 GPa) as a standard.

## 3. Results

**Figure 1:**
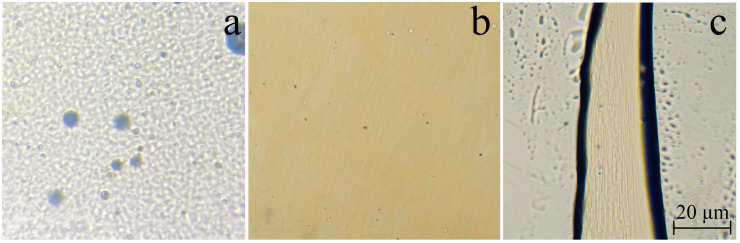
Morphology of an 18% mixed solution in NMMO with a polymer ratio of 70% PAN/30% cellulose (T = 120 °C): under stationary conditions (**a**) and under shear (**b**,**c**).

To obtain a fibrillar structure in composite fibers, 18% mixed solutions in NMMO containing 70% PAN and 30% cellulose were utilized. The morphology of the mixed solution placed between glasses (T = 120 °C) is shown in Figure 1.

After intensive mixing of a two phase emulsion consisting of a PAN solution in NMMO and a cellulose solution in NMMO, the resulting morphology is difficult to separate into a dispersed medium and a phase (Figure 1a). Component solutions have a close to uniform distribution in volume. However, with a slight shift of the surface glass relative to the object glass, the morphological picture alters. The cellulose solution is elongated along the deformation axis, forming thin rod-like structures in the volume of the dispersed media of the PAN solution (Figure 1b). When the mixture solution is forced through the viscometer’s capillary, fibrils are formed (Figure 1c). The difference in viscosity between the dispersed medium and phase solutions should assist in their formation. According to [55], the ratio of the viscosities of the dispersed medium and the phase should be close to unity for maximum fibrillization, as demonstrated in the work [56]. Another example is that the viscosity of the dispersed medium must be greater than that of the dispersed phase in order for fibrils to develop [57]. For the system under consideration, the viscosity at 1 s^−1^ is 36 Pa*s (T = 120 °C). The viscosity of a 12.6% solution of PAN in NMMO is 0.54 Pa*s, and that of a 5.4% solution of dispersed cellulose phase is 24.4 Pa*s. Thus, the viscosity ratio of the matrix solution is 45 times lower than that of the dispersed phase. The viscosity of the mixed solution is 1.5 times higher than that of the more viscous cellulose phase. An explanation for fibril formation was suggested based on the interaction of PAN and the cellulose phase [42].

**Figure 2:**
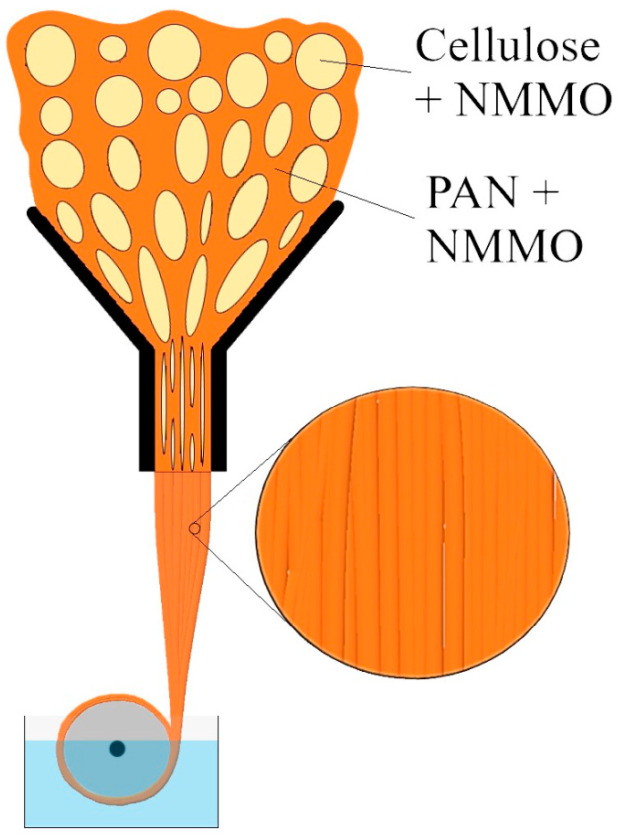
Schematic representation of a mixed PAN-cellulose solution passing through a viscometer’s capillary.

During the process of forcing the mixture solution through the viscometer’s capillary, the dispersed phase is subjected to longitudinal deformation at the entrance to the die channel and shear deformation when passing through it (Figure 2).

It is known that in the process of moving similar systems through a die, the formation of various morphological patterns is possible. In this case, fibrils may form at the capillary channel’s entrance, followed by destruction or change within it or at its departure. It should be noted that raising the fiber drawing ratio produces thinner cellulose fibrils.

**Figure 3:**
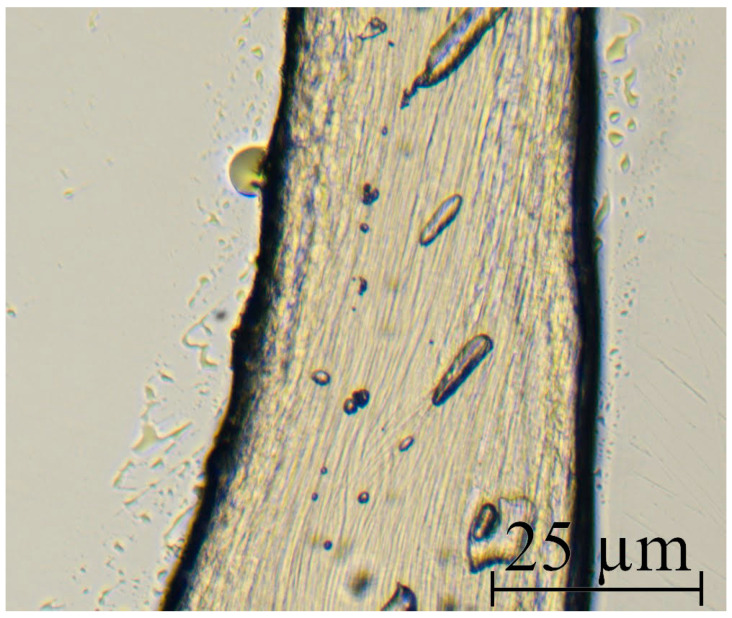
Morphology of a just spun non-precipitated extrudate formed from an 18% blend solution in NMMO (70% PAN/30% cellulose, T = 120 °C).

The morphology of the non-precipitated just-spent extrudate was discussed before (Figure 1c). The question arises if the fibrillar structure will be preserved when the formed system is deformed. To answer this question, the unprecipitated sample was placed between glasses at T = 120 °C and subjected to shear deformation (Figure 3).

When pressure is applied to the cover glass, the extrudate becomes deformed and increases its width. The formed fibrils, moving relative to each other, practically do not change their appearance. The thickness of the fibrils is up to several microns. Visually, their thickness remains unchanged when the extrudate is deformed. After the stress is removed, the extrudate partially restores its appearance.

**Figure 4:**
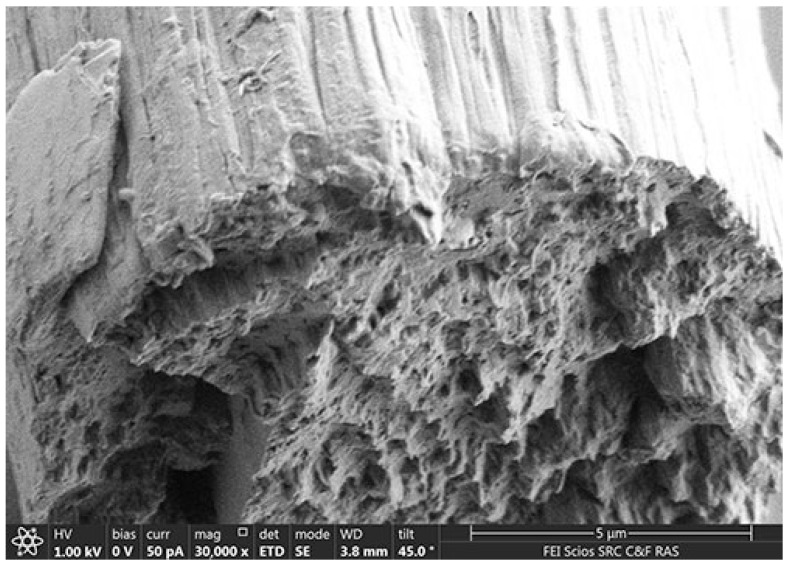
Microphotographs of 60% PAN-40% cellulose fiber.

When forming fibers using the dry-jet wet method, the jet solution emerging from the capillary bypasses the air gap and enters the precipitation bath. The polymer phases begin to coagulate after coming into contact with the bath’s water. The difficulty of coagulating a two-phase system lies in the sequential separation of the PAN matrix polymer and then the fibrillar cellulose phase. After completely removing the solvent from the system and drying the fibers, their morphology was examined using scanning microscopy (Figure 4).

The composite fiber’s surface is best compared to the morphology observed for cellulose or PAN fibers. These fibers are characterized by a smooth, defect-free morphology. In contrast, the composite fiber’s surface is not smooth and reflects a fibrillar internal morphology.

The transverse cleavage of PAN and Lyocell fibers mimics a circle and has a monolithic (dense) structure. For composite fibers, despite the wavy surface texture, the transverse cleavage also resembles a circle. The average diameter of Lyocell, PAN, and composite fibers varies from 12 to 17 microns.

Lyocell (Orcel) fibers have crystalline and amorphous areas oriented longitudinally, resulting in fibrils smaller than 0.1 μm. The fibrils are packed so densely that they do not appear in fiber cross-sections. Composite fibers have a different morphology; they are characterized by a larger heterogeneous texture formed by microfibers of the order of 1 μm rather than fibrils. Microfibers are also heterogeneous, consisting of many oriented fibrillar subunits. The surface of the fiber is grooved due to microfibers protruding from the surface, but the cross-section is usually circular.

The strength and deformation properties of fibers are important markers of their quality. In this study, composite fibers’ mechanical properties were compared to those of PAN and Lyocell fibers (Table 1).

The diameters of the studied fibers range from 10.8 to 19.3 microns, which is similar to the sizes of industrial samples. Lyocell fibers’ dry strength can exceed 500 MPa and reach 1 GPa [58]. The great strength of Lyocell fibers is due to the high orientation of cellulose macromolecules formed during fiber spinning [59]. The highest strength of wood cellulose fibers was 630 MPa, while PAN fibers had a strength several times lower. The introduction of cellulose into the PAN matrix slightly increases the composite fibers’ strength and elastic modulus. In this case, the elongation at break is reduced to values characteristic of cellulose fibers (PAN fibers were not subjected to thermal drawing).

**Figure 5:**
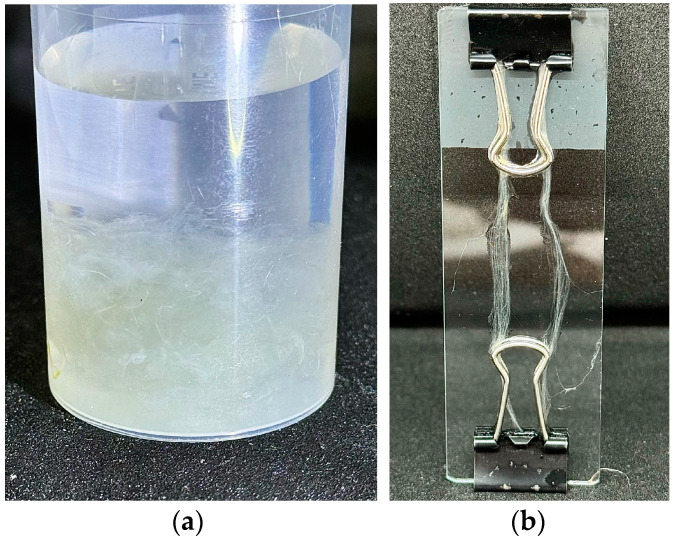
Composite fibers with 70% PAN-30% cellulose in DMSO (**a**) and isolated cellulose microfibers (**b**).

Thus, the introduction of a cellulose “reinforcing” phase in the form of fibrils into the PAN matrix does not lead to any significant increase in strength or elastic modulus. In contrast, the addition of cellulose significantly reduces the deformation properties of composite fibers. The question arises: what influences the strength and deformation properties of composite fibers: the matrix or the filler? To answer this question, PAN was removed by selective dissolution using DMSO. It is known that cellulose is not soluble in DMSO, and its properties change slightly when kept in this non-solvent. Figure 5 shows a photograph of fibers being washed from PAN.

The initial composite fibers were a skein of parallel monofilaments with a diameter of 12 cm. They were light-brown in color and eventually converted to light-yellow and white when PAN was removed. Removal of PAN for thinner fibers was faster compared to thicker ones, as observed visually. To obtain parallel microfibers, composite samples were preliminarily fixed into frames with a length of at least 3–5 cm (Figure 5b). The isolated cellulose microfibers were washed with distilled water and dried at room temperature to a constant weight.

**Figure 6:**
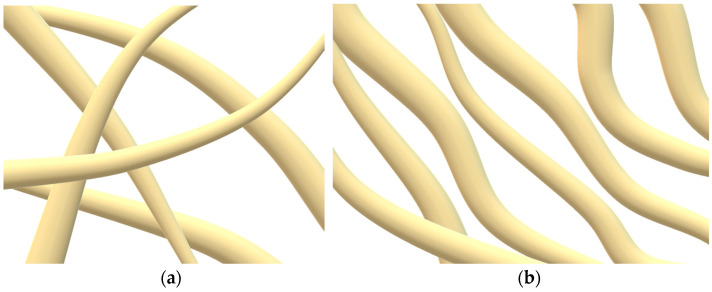
Scheme of the arrangement of fibers during the isolation of the cellulose phase without (**a**) and with fixation (**b**).

When the separated cellulose fibers were not fixed, a suspension containing densely intertwined microfibers was formed (Figure 6).

The arrangement of cellulose microfibers determines their future use. In the case of chaotic interweaving of microfibers (Figure 6a), the formed mesh allows the formation of nonwoven materials or paper of minimal thickness. Parallel fibers can be used to produce multifilament yarns, reinforce polymer materials, weave, and so on.

**Figure 7:**
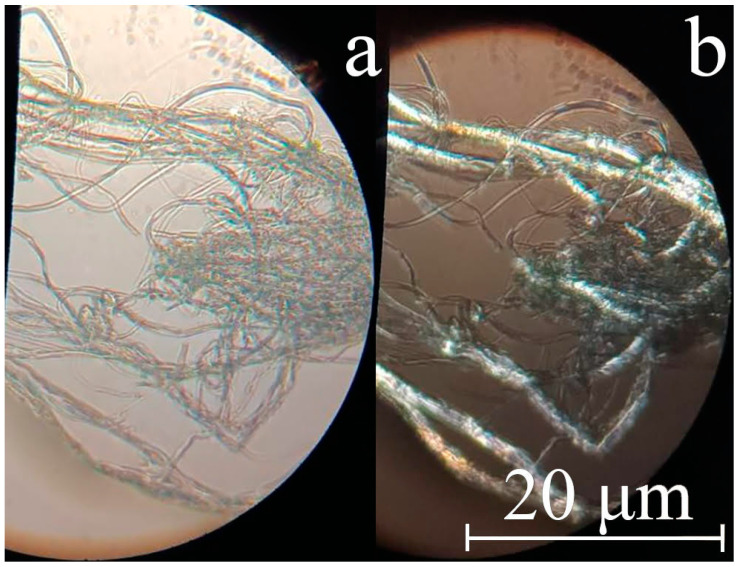
Photographs of cellulose microfibers after removal of PAN: in transmitted light (**a**) and crossed polaroids (**b**).

**Figure 8:**
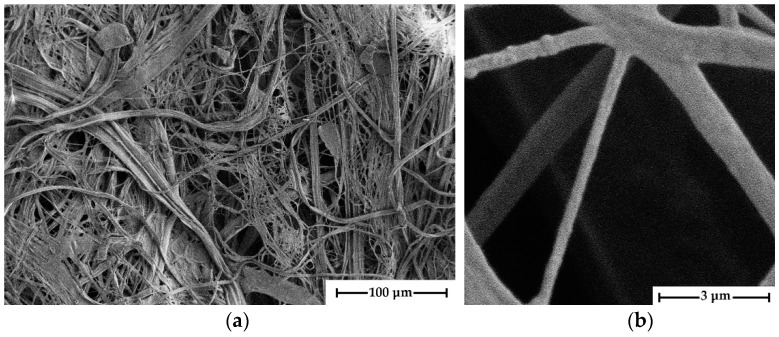
SEM micrographs of microfibers isolated from composite fibers of the composition 70% PAN-30% cellulose at various magnification levels: ×1049 (**a**) and ×50,000 (**b**).

The morphology of cellulose microfibers was studied with optical polarization microscopy (Figure 7), scanning electron microscopy (Figure 8), and atomic force microscopy.

The individual cellulose microfibers can have a diameter of up to several microns. For the most elongated composite fibers, the microfiber diameter does not exceed 1–2 microns. Microfibers intertwine with each other and form twisted “threads” of micron and submicron diameter.

The fibers in crossed polaroids have a cellulose glow, which means that during the precipitation process, a crystalline phase forms in the separated microfibers. It is interesting that the cellulose phase in the composite system separates after the coagulation of PAN. Because of its intense contact with the solvent, the precipitant must diminish its activity after reaching the composite fiber’s volume. Simultaneously, a decrease in the rate of its diffusion should be observed. When the cellulose phase is reached, the water-NMMO mixture system promotes soft cellulose precipitation. Such precipitation usually results in a more homogeneous structure [60,61]. When using a rigid precipitant, on the other hand, a defective morphology is observed that includes vacuoles of varied lengths and widths, pores, surface cracks, heterogeneity of the fiber’s cross-section, and so on. Previous research has shown that the precipitation number can be used as a parameter characterizing the degree of coagulation of a polymer solution. The precipitation number is the amount of precipitant needed (in ml) to induce the system to disintegrate into phases (polymer release) and change the optical properties of the solution (turbidity). 1% PAN solutions in NMMO require substantially less precipitant (water) than solutions in solvents such as DMSO, DMF, and so on [62], as well as cellulose solutions in NMMO [63]. According to [64], low precipitation threshold values imply a strong affinity of the precipitant for the solvent.

The given micrographs show that the diameter of the fibers varies widely, ranging from tens of nanometers to several micrometers. The thickness of the microfibrils depends on the drawing ratio of the formed fiber, and with its increase, the diameter of the cellulose fibers decreases. During the process of removing PAN with DMSO in a free state, cellulose microfibrils form a dense network that resembles that of nonwoven materials. The interweaving and entangling of cellulose microfibrils occurs in the volume of the selective solvent PAN, followed by water, which replaced DMSO. Following drying, the mesh of interlocking “nonwoven” micromaterial is supplemented with dispersion forces, which, when combined with frictional forces, provide good strength to cellulose samples. The stated structure is interesting for producing nonwoven materials, filters, and membranes.

**Figure 9:**
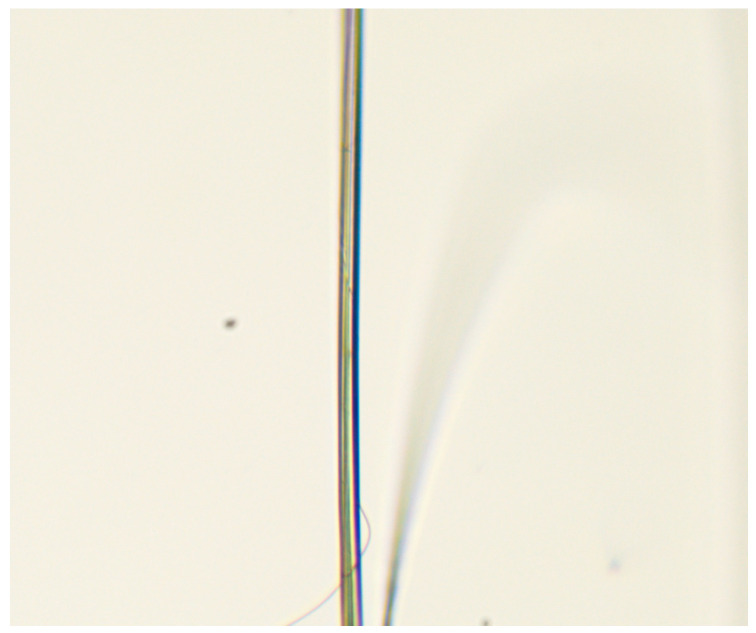
Photograph of isolated cellulose microfiber, fiber diameter 3 µm.

The fibrils have a smooth surface with no noticeable flaws (crazes, cracks, or depressions). To determine the mechanical properties of microfibers, they were isolated when wet. Following that, the fibers were fixed in a frame and conditioned at room temperature. Figure 9 shows a photograph of an individual microfiber.

All isolated microfibers have smooth and even edges, and the fiber thickness varies slightly from the observed average value along its whole length. Unfortunately, thinner fibers cannot be isolated because they quickly agglomerate due to the presence of several OH-groups on their surface, which contribute to the creation of hydrogen bonds. Once such strong bonds are formed, it is almost impossible to separate microfibrils without breaking even in water.

The strength of the cellulose microfibers reached 350 MPa, which was lower than that of Lyocell fibers but nearly doubled that of PAN and composite fibers. The elastic modulus of cellulose microfibers (32.8 GPa) exceeds all known values for Lyocell, PAN, and composite fibers. The elongation of microfibers varies from 5.3 to 9.2%, which is close to the values for Lyocell and composite fibers and several times less compared to PAN fibers. The mechanical properties of cellulose microfibers show that the microfibrillar phase influences both the strength and deformation characteristics of composite fibers. Cellulose acts as a reinforcing phase, retaining the rigidity of Lyocell fiber.

**Figure 10:**
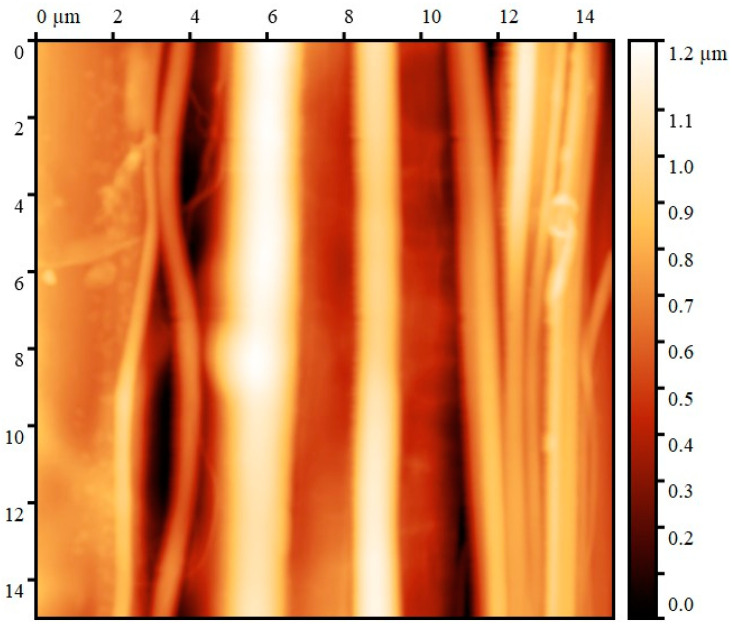
Topographic map of isolated cellulose microfibers.

The elastic modulus values were also determined using atomic force microscopy. Using the PeakForce-QNM method, topographical (relief) (Figure 10) and Young’s modulus maps were simultaneously obtained.

The given topographic maps show that the average diameter of the fibers is not more than a few microns. The margins of the fibers are smooth, and the measured diameter varies little along the fiber axis.

**Figure 11:**
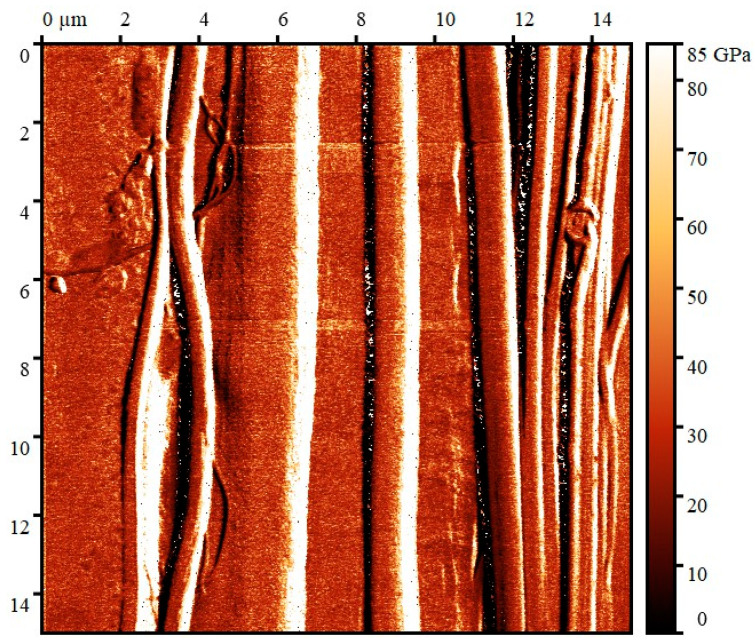
Map of Young’s modulus values for cellulose microfibers.

Figure 11 shows a map of Young’s modulus values corresponding to the resulting topographic map.

For quantitative measurements, a relative calibration method was utilized with a fused quartz sample (E = 73 GPa) as a standard. The average Young’s modulus in the analyzed areas is from 20 to 33 GPa. Also, Young’s modulus was studied using the ForceVolume mode, which has a much lower probing rate and thus lower speed and lower resolution; the obtained values are in good correlation with the values presented above.

It should be noted that in areas of the scan where there is a large surface slope (more than 10°), the Young’s modulus is greatly overestimated due to slippage of the probe [65]; these are the areas colored white on the Young’s modulus map.

It is known that the hydrophilic nature of cellulose limits the applicability of cellulose fibrils for the production of nanocomposites where synthetic polymers act as a matrix. This is due to the difficulties of dispersing and introducing cellulose into the matrix. The introduction of cellulose into a synthetic matrix is often accompanied by the formation of defects in the molded material in the form of voids as well as particle agglomerates. On the other hand, cellulose is capable of forming various complex geometric shapes, including elongated particles with a high form factor. This means that such particles can promote the orientation of molecules in the polymer matrix and increase the strength of the composite. Cellulose particles have high strength, rigidity, and low density, which provide high interest in such a dispersed filler.

**Figure 12:**
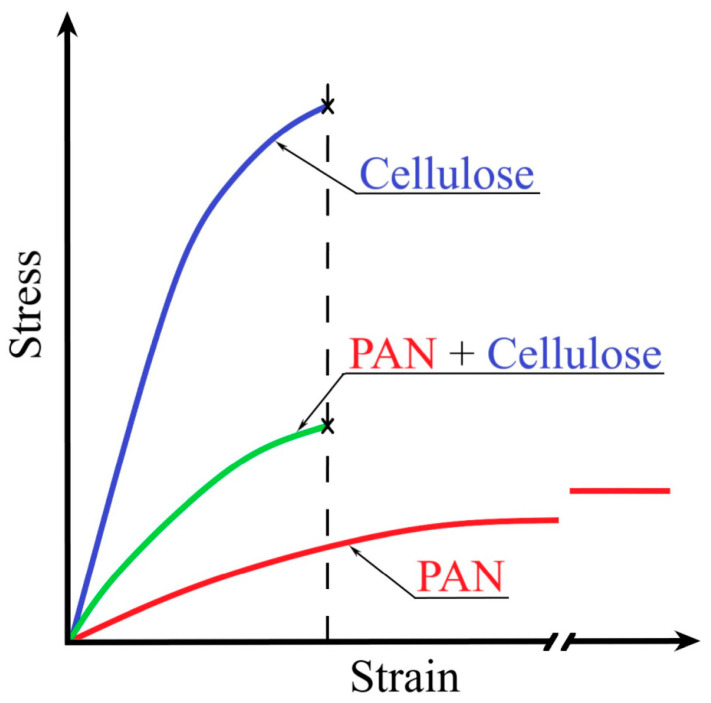
Stress-strain curves for cellulose, PAN, and composite fibers.

As previously stated, the filler with the highest form factor is the most preferable among all types of cellulose. The introduction of such filler into the polymer matrix will increase the strength of the composite. Typically, the matrix, filler, and composite deformation curves appear as shown in Figure 12.

At maximum relative elongation, PAN fibers exhibit low strength and modulus values. In contrast, cellulose fibers have substantially lower elongation but the highest strength and modulus. Composite fibers exhibit intermediate mechanical properties. Their deformation properties are similar to those of cellulose fibers, but their strength and elastic modulus are comparable to those of PAN fibers.

The introduction of filler into the polymer matrix with the long length obtained in this study is quite challenging. An alternative method is to generate an ordered dispersion phase during the spinning process. The mechanical properties of microfibrils have been found, allowing us to take a fresh look at the mechanical properties of composite fibers. We can discuss solving the inverse problem, generating composite fibers, and then assessing the mechanical properties of the filler.

Cellulose microfibrils are as strong as Lyocell fibers, and their elastic modulus occasionally exceeds comparable values. The lower strength values for composite fibers are most likely attributable to the poor orientation of the PAN matrix. PAN fibers typically have strength after spinning ranging from 100 to 200 MPa, which increases dramatically when thermally drawn. The rigid coagulation conditions of composite fibers also influence their mechanical properties. The PAN matrix solution precipitates first, followed by the cellulose phase under more soft conditions. Increasing the drawing ratio and adjusting the PAN/cellulose ratio in the future may allow for the production of a more advanced microfibrillar structure while also increasing the strength of the cellulose phase and composite fibers.

## 4. Conclusions

When mixed solutions of PAN and cellulose in NMMO are passed through a capillary, various morphologies can be formed. The type of morphology formed will be determined by the channel’s shape, the intensity of solution deformation, and the polymer ratio in the system. When mixed solutions with a PAN content of 60% or above are extruded, fibrillar morphology develops. The dispersed phase of a cellulose solution in NMMO forms extended microfibrils. Composite fibers retain the fibrillar shape that is generated during coagulation. Composite fibers have slightly higher strength and modulus values than PAN fibers; nevertheless, relative elongation values have dropped sharply to levels similar to those of cellulose fibers. This enabled us to propose a hypothesis on the highly significant role of the cellulose phase in the mechanical properties of composite fibers. Isolating cellulose microfibers through selective dissolution of a PAN matrix allowed for the determination of their mechanical properties. Thus, the strength of microfibers reaches 350 MPa, the elastic modulus is 32 GPa, and the relative elongation is 8%. This study confirmed the hypothesis that the fibrillar shape of the cellulose phase in mixed solutions might be used to change the mechanical characteristics of PAN fibers. At the same time, the strength of cellulose microfibers is 1.8 times greater compared to PAN fibers. The presented results open up new possibilities for the controlled production of cellulose microfibers from concentrated mixed solutions. The structure, morphology, and properties of the resulting fibrils will depend on the conditions of their preparation, in particular, on the conditions for the separation of the polymer phase from the solution; future work will be devoted to these issues. The isolated microfibers can subsequently be utilized to make filters, membranes, reinforcing materials, and low-density fabrics.

## Figures and Tables

**Table 1:** Mechanical properties of PAN, Lyocell, and composite fibers.

Sample	Diameter, μm	Tensile Strength, MPa	Modulus of Elasticity, GPa	Elongation, %
100% cellulose	12.1–18.9	410–630	7.2–19.6	5–9
100% PAN	12.2–19.3	96–200	2.1–4.1	60–130
70% PAN-30% cellulose	10.8–13.8	96–250	2.4–5.4	4.5–8.1

## Data Availability

The original contributions presented in the study are included in the article, further inquiries can be directed to the corresponding author.
